# Twelve exonic variants in the *SLC12A1* and *CLCNKB* genes alter RNA splicing in a minigene assay

**DOI:** 10.3389/fgene.2022.961384

**Published:** 2022-08-25

**Authors:** Qing Xin, Qihua Liu, Zhiying Liu, Xiaomeng Shi, Xuyan Liu, Ruixiao Zhang, Yefeng Hong, Xiangzhong Zhao, Leping Shao

**Affiliations:** ^1^ Department of Nephrology, the Affiliated Qingdao Municipal Hospital of Qingdao University, Qingdao, China; ^2^ Department of Material Supply Management, the Affiliated Qingdao Municipal Hospital of Qingdao University, Qingdao, China; ^3^ Department of Cardiology, the Affiliated Hospital of Qingdao University, Qingdao, China; ^4^ Medical Research Center, The Affiliated Hospital of Qingdao University, Qingdao, China

**Keywords:** *SLC12A1* gene, *CLCNKB* gene, pre-mRNA splicing, exonic mutation, exon skipping

## Abstract

**Background:** Bartter syndrome (BS) is a rare renal tubular disease caused by gene variants in *SLC12A1*, *KCNJ1*, *CLCNKA*, *CLCNKB*, *BSND* or *MAGED2* genes. There is growing evidence that many exonic mutations can affect the pre-mRNA normal splicing and induce exon skipping by altering various splicing regulatory signals. Therefore, the aim of this study was to gain new insights into the consequences of exonic mutations associated with BS on pre-mRNA splicing.

**Methods:** We analyzed all the missense, nonsense and synonymous variants described in six pathogenic genes by bioinformatics programs and identified candidate mutations that may promote exon skipping through a minigene system.

**Results:** Results of the study showed that 12 of 14 candidate variants distributed in *SLC12A1* (c.728G>A, C.735C>G, c.904C>T, c.905G>A, c.1304C>T, c.1493C>T, c.2221A>T) and *CLCNKB* (c.226C>T, c.228A>C, c.229G>A, c.229G>C, c.1979C>A) were identified to induce splicing alterations. These variants may not only disrupt exonic splicing enhancers (ESEs) but also generate new exonic splicing silencers (ESSs), or disturb the classic splicing sites.

**Conclusion:** To our knowledge, this is a comprehensive study regarding alterations in pre-mRNA of exonic variants in BS pathogenic genes. Our results reinforce the necessity of assessing the consequences of exonic variants at the mRNA level.

## Introduction

Pre-messenger RNA splicing is an essential and precisely controlled process by which noncoding introns are removed and exons are ligated together to produce mature mRNAs (Marengo and Wassarman, 2008). Splicing is carried out by a macromolecular complex that is known as the spliceosome, composed of five small nuclear ribonucleoproteins (snRNPs, U1, U2, U4, U5, U6) and several corresponding protein factors (Warf and Berglund, 2010). The spliceosome assembles at the exon-intron junctions and interacts with splice-sites to catalyze the splicing reaction (Brow, 2002; Patel and Steitz, 2003; Chen and Manley, 2009).

Altered pre-mRNA splicing has long been known as the underlying cause of rare hereditary disorders (Dhayat et al., 2016; Scotti and Swanson, 2016; Suarez-Artiles et al., 2018). In addition to the well-known mechanism that intron mutations can affect the splicing process by directly altering splicing sites (5′ donor and 3’ acceptor splicing sites, and branch sites), more and more studies have shown that mutations in exons can also interfere with precursor RNA splicing and promote the activation of new cryptic splice sites by affecting a variety of splicing auxiliary signals (Gonzalez-Paredes et al., 2014). Furthermore, exons contain many splicing regulatory elements, such as exonic splicing enhancers (ESEs) and exonic splicing silencers(ESSs), which facilitate or repress the identification of splice-sites (Cartegni et al., 2002). Serine/arginine (SR) proteins and heterogeneous ribonucleoproteins (hnRNPs) families are most well-known trans-factors that exert function by interacting with these elements (Martinez-Contreras et al., 2007; Long and Caceres, 2009). The pre-mRNA splicing process will be changed when these splicing signals are newly created or deactivated by some point mutations (Blencowe, 2000; Claverie-Martin et al., 2015).

Bartter Syndrome (BS), initially described in 1962, is a group of autosomal recessive inherited renal disorders characterized by hypokalemia, hypochloremia and metabolic alkalosis, normal blood pressure with elevated renin and aldosterone levels (Bartter et al., 1962). To date, BS can be further subdivided into 5 different subtypes according to their causative genes: BS I (*SLC12A1*), BS II (*KCNJ1*), BS III (*CLCNKB*), BS IV (*BSND, CLCNKA* and *CLCNKB*) and BS V(*MAGED2*) (Kleta and Bockenhauer, 2006; Walsh et al., 2018; Besouw et al., 2020). The *SLC12A1* gene is located on chromosome 15q21.1 with 29 exons and encodes the bumetanide-sensitive Na^+^-K^+^-2Cl^-^ cotransporter (NKCC2) (Kemter et al., 2014). Bartter syndrome type 3 is caused by mutations in *CLCNKB* gene, which is found on chromosome 1p36.13 with 20 exons and encodes the ClC-Kb chloride channel protein (Seys et al., 2017). Efficient functioning of these encoded proteins is crucial for renin release and tubuloglomerular feedback. Mutations will probably induce the formation of abnormal proteins, leading to severe electrolyte abnormalities and refractory arterial hypotension (Seaayfan et al., 2016; Wang C. et al., 2020). In addition, mutations associated with the other four genes are also implicated in the pathogenesis of BS.

Among all the BS-related variants described in the Human Gene Mutation Database (HGMD, accessed February 2021), there are 209 missense mutations or nonsense mutations, 21 splice site mutations, 40 small deletions, 16 small insertions, 19 gross deletions and 2 complex rearrangements. We noted that missense and nonsense mutations accounted for 68%(209/307)of all the mutations. Up to now, most variation analyses are performed merely at genome level. Therefore, it would be interesting to investigate the potential effect of missense and nonsense mutations at both DNA and RNA levels (Suarez-Artiles et al., 2018; Wang S. et al., 2020; Zhang et al., 2021).

Since the impact of exonic mutations of pathogenic genes in BS on pre-mRNA splicing remains poorly investigated, we aimed to provide deeper insights using bioinformatics tools and minigene assays in this study.

## Materials and methods

### Variant nomenclature

The nomenclature of mutations followed the guidelines of the Human Genome Variation Society (http://varnomen.hgvs.org). DNA mutation numbering was based on the cDNA sequence for *SLC12A1* (RefSeq NM_000338.3), *KCNJ1* (RefSeq NM_000220.6), *CLCNKA* (RefSeq NM_001042704.2), *CLCNKB* (RefSeq NM_000085.5), *BSND* (RefSeq NM_057176.3) and *MAGED2* (RefSeq NM_014599.6).

### In silico analyses and screening criteria

All missense, synonymous and nonsense variants associated with genes in BS were selected from the HGMD (February 2021) and literature (Wongsaengsak et al., 2017; Daga et al., 2018; Elrharchi et al., 2018; Wang et al., 2018; Li J. et al., 2019; Li Y. et al., 2019; Xue et al., 2019), except 21 variants that were identified and analyzed in our previous study (Han et al., 2017).

To evaluate the potential effects of missense variants on the protein level, the following prediction software were used: Polymorphism Phenotyping v2 (http://genetics.bwh.harvard.edu/pph2/), MutationTaster (https://www.mutationtaster.org/) and Provean (http://provean.jcvi.org/protein_batch_submit.php?species=human).

In silico analysis were performed through online bioinformatics software in order to determine their potential effects on pre-mRNA processing and predict protein alterations following splicing defects. To identify the presence of putative splicing regulatory sequences, and determine potential effect of variations on splicing regulatory motifs (ESEs/ESSs), we used online software Human Splicing Finder (https://www.genomnis.com/access-hsf). Additionally, for the mutations close to the 5′ or 3′ ends of exons, we also performed BDGP (http://www.fruitfly.org) and MaxEntScan (http://hollywood.mit.edu/burgelab/maxent/Xmaxent.html) evaluation to analyze the effect of variants on 5′donor or 3′acceptor consensus sites and to predict the generation and/or activation of novel sites. For experimentally validated sites that alter exon splicing, we used SnapGene software and UniProt (https://www.uniprot.org/) to perform predictive analysis of reading frame changes and following protein defect.

For the experimental analysis of our study, criteria for selecting the mutations were as follows: 1) close to the 5′ or 3′ ends of exons. 2) predicted elimination of ESEs or new creation of ESSs.

### Minigene constructions and site-directed mutagenesis

**FIGURE 1:**
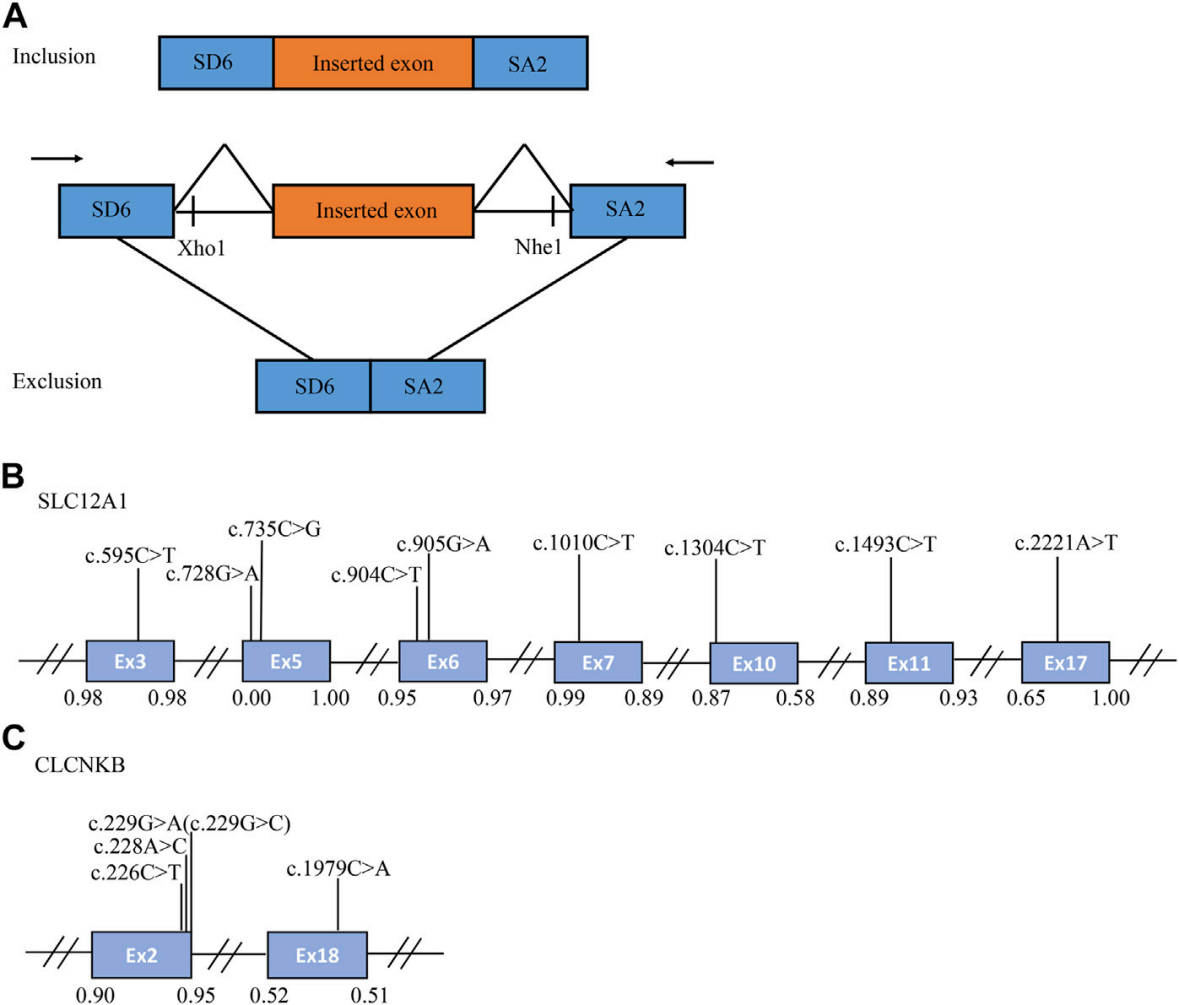
The schematic diagram of minigene based on the pSPL3 exon trapping vector and position of 14 presumed exonic variants. **(A)** Wild-type and mutant fragments of target exon, flanked by part of the upstream and downstream intronic sequence, were separately cloned into the XhoI and NheI cloning sites of the pSPL3 vector **(B,C)** Position of presumed exonic variants in *SLC12A1* and *CLCNKB* gene. Blue boxes and black lines between them represent the coding exons and introns sequences, respectively. Their sizes are not proportional. The BDGP scores of donor and acceptor splice sites are represented in decimal.

A minigene splicing assay was performed to validate whether these variants affect the splicing process. Constructions of minigene have been described previously (Zhang et al., 2021). For the *in vitro* splicing assay, the fragments involving target exon, flanked by approximately 50–200 nucleotides of shortened introns, were amplified by PCR reactions and specific oligonucleotide primers. These primers have XhoI and NheI restriction sites (XhoI: CCGC ˆ CTCGAG; NheI: CTAG ˆ CTAGC) to facilitate cloning into the splicing vector pSPL3 (Figure 1A). The design of primers was done according to the procedure of Primer BLAST, and the sequence of primers were shown in Supplementary Table S1. All PCR products were purified with Gel Extraction kit (Cwbio, China).

The expression vector and the insert DNA were ligated at 16°C using 1ul of T4 DNA Ligase (Takara, Japan) and transformed into DH5α competent E. coli cells (Zhang Q. et al., 2018). Two-hundred microliters of bacteria suspension was spread evenly on the solid ampicillin-Luria-Bertani agar plates and coated for 16h at 37°C. The positive clones were screened and sequenced using forward and reverse primers, the sequencing results were shown in Supplementary Figure S1. Snapgene software was used for sequence alignment analysis. The collected monoclonal colonies were extracted using Pure Plasmid Mini Kit (Cwbio, China).

Variants of interest were introduced into exons with Quik Change II Site Directed Mutagenesis Kit (Stratagene, La Jolla, CA, United States) as instructed by the manufacturer. Mutagenesis primers were designed by Snapgene and Primer BLAST and listed in Supplementary Table S2. In order to confirm the existence of target mutations, all minigenes were further transformed into DH5α-competent cells and screened by conventional sanger sequencing. Primer extension reactions were set up as follows: the first step is denaturation at 98°C for 10 s, annealing at 62°C for 10 s, elongation at 72°C for 45 s, followed by 20 cycles, and finally extension at 72°C for 2 min.

### Transfection of HEK 293T and hela cells

Human embryonal kidney 293T (HEK293T) and Hela cells were cultured in DMEM medium containing high glucose (4.5 g/L), supplemented with 10% fetal bovine serum at 37°C in a 5% CO_2_ humidified incubator. One day before transfection, cells were seeded on a 24-well plate to grow to 70–80% confluence in an antibiotic-free medium. Transfections of each group of minigenes (empty pSPL3 control, wild and mutant type) were carried out using Lipofectamine 3,000(Invitrogen, United States) following the manufacturer’s instructions.

### Minigene splicing assay

After 48h incubation, total RNA was extracted using TRIzol reagent (Invitrogen, United States). cDNA synthesis was performed with PrimeScript 1st Strand cDNA Synthesis kit (Takara, Japan) (Shao et al., 2018). The PCR amplification reaction of cDNAs was carried out with primers: SD6(the forward primer: 5′-TCT​GAG​TCA​CCT​GGA​CAA​CC-3′) and SA2 (the reverse primer: 5′-ATC​TCA​GTG​GTA​TTT​GTG​AGC-3′). The PCR procedures were as follows: in 20 μL volume, 2 μL of cDNA, 10 μL of 2×Tap Plus Master Mix (Vazyme, China), 1 μL of each primer, and 6 μL ddH_2_O. Thermal conditions were denaturation at 95°C for 3 min, 7 cycles of 95°C for 15 s, 62°C for 20 s, and 72°C for 30 s, 8 cycles of 95°C for 15 s, 60°C for 20 s, and 72°C for 30 s, 8 cycles of 95°C for 15 s, 58°C for 20 s, and 72°C for 30 s, and followed by a final elongation step at 72°C for 5 min. PCR products are then resolved by 1.5% agarose gel electrophoresis. Signal intensity of each band was quantified using the software ImageJ. DNA bands with the correct size were cut out and purified using a Gel Extraction Kit (Cwbio, China). All transcripts were sequenced as previously described.

The Snapgene software was used to compare our gene sequences with the reference sequences published in GenBank. If the splicing pattern was different from the WT minigene both in HEK 293T and Hela cells, variation was believed to contribute to splicing defects.

### Statistical analysis

The percentage of the abnormal splicing was densitometrically calculated as (lower band/[lower band + upper band]) × 100. The error bars represent SEM (n = 3). *p < 0.05, unpaired Student’s t-test.

## Results

A total of 170 variants (72 in *SLC12A1*, 74 in *CLCNKB*, 18 in *BSND*, 3 in *CLCNKA*, 3 in *MAGED2*) were compiled from database and analyzed using bioinformatics software. The variants c.2T>G and c.2017A>T in *CLCNKB* were eliminated since they were located in the first and last exon respectively and therefore could not be analyzed by minigene approach. We excluded *KCNJ1* gene because it had only two exons. Finally, we selected potential splicing variants within 4 bases of 5′ or 3’ end of the exons and that were predicted to affect splicing regulatory elements (the total number of broken ESEs and gained ESSs is more than 5) for further investigation (Mertes et al., 2021). Combined with the above analysis, the enrolled variants were as follows: 9 in *SLC12A1* (c.595C>T, c.728G>A, c.735C>G, c.904C>T, c.905G>A, c.1010C>T, c.1304C>T, c.1493C>T, c.2221A>T), and 5 in *CLCNKB* (c.226C>T, c.228A>C, c.229G>A, c.229G>C, c.1979C>A) as shown in Table 1 and Figures 1B,C. In addition, nine of these mutants were missense, therefore we performed a protein-level predictive analysis on them, and details are listed in Table 2. Most of the mutants were predicted to be “pathogenic”, except for c.1304C>T, which was predicted to be “benign” by polyphen2. Therefore, it would be interesting to explore the potential different effects of these mutants at the RNA level.

**TABLE 1:** Variants selected from this study in *SLC12A1* and *CLCNKB*, and the results of silico analyses.

Gene	Mutation		Exon (length)	Location in exon	BDGP	New ESS Site	ESE Site Broken	△MaxEnt Donor site	△ MaxEnt Acceptor site	References
*SLC12A1*	c.595C>T	p. Arg199Cys	3(76)	−34	NA	6	1			Ji et al. (2008)
*SLC12A1*	c.728G>A	p. Gly243Glu	5(140)	+4	3′AS: 0.99→0.98	0	0			Vargas-Poussou et al. (1998)
*SLC12A1*	c.735C>G	p. Tyr245*	5(140)	+11	NA	1	5			Wongsaengsak et al. (2017)
*SLC12A1*	c.904C>T	p. Arg302Trp	6(111)	+40	NA	1	6			Ji et al. (2008)
*SLC12A1*	c.905G>A	p. Arg302Gln	6(111)	+41	NA	2	6			Vargas-Poussou et al. (1998)
*SLC12A1*	c.1010C>T	p. Ala337Val	7(112)	+35	NA	6	2			Brochard et al. (2009)
*SLC12A1*	c.1304C>T	p. Ala435Val	10(152)	+4	3′AS: 0.87→0.84	2	2			Han et al. (2019)
*SLC12A1*	c.1493C>T	p. Ala498Val	11(108)	+41	NA	5	2			Puricelli et al. (2010)
*SLC12A1*	c.2221A>T	p. Lys741*	17(141)	+67	NA	2	4		2438.46%	Han et al. (2019)
*CLCNKB*	c.226C>T	p. Arg76*	2(129)	−4	5′DS: 0.95→0.93	4	1			Nozu et al. (2010)
*CLCNKB*	c.228A>C	p.76Arg =	2(129)	−2	5′DS: 0.95→0.84	0	0	−36.73%		Wang et al. (2020a)
*CLCNKB*	c.229G>A	p. Ala77Thr	2(129)	−1	NA	0	0	−73.68%		Konrad et al. (2000)
*CLCNKB*	c.229G>C	p. Ala77Pro	2(129)	−1	NA	0	0	−55.79%		Robitaille et al. (2011)
*CLCNKB*	c.1979C>A	p. Ser660*	18(87)	−38	NA	3	5		410.71%	Han et al. (2020)

Location of 14 variants: “ + ” indicates distance from the 5′ end of the exon and “−” represents distance from the 3′ end. AS, acceptor splice sites; DS, donor splice sites. Value 0, 1, 2, 3, 4, 5, and 6 indicate number of the splicing regulatory elements gained or disrupted. ESE, exonic splicing enhancer; ESS, exonic splicing silencer; NA, not applicable. ΔMaxEnt = MaxEnt Var −MaxEnt WT < 0 are assumed potential loss of 5′ donor or 3′ acceptor splice-site.

**TABLE 2:** Prediction of the potential pathogenicity of the missense variants.

Gene	Variants	Mutation Taster (score)	Provean (cutoff = -2.5)	SIFT (cutoff = 0.05)	PolyPhen-2 (score)
*SLC12A1*	c.595C>T	Disease causing(0.9999)	Deleterious −7.45	Damaging 0.000	PROBABLY DAMAGING 1.000
*SLC12A1*	c.728G>A	Disease causing(0.9999)	Deleterious −7.51	Damaging 0.000	PROBABLY DAMAGING (1.000)
*SLC12A1*	c.904C>T	Disease causing(0.9999)	Deleterious −7.56	Damaging 0.000	PROBABLY DAMAGING (1.000)
*SLC12A1*	c.905G>A	Disease causing(0.9999)	Deleterious −3.78	Damaging 0.001	PROBABLY DAMAGING (1.000)
*SLC12A1*	c.1010C>T	Disease causing(0.9999)	Deleterious −3.40	Damaging 0.001	PROBABLY DAMAGING 0.975
*SLC12A1*	c.1304C>T	Disease causing(0.9999)	Deleterious −2.87	Damaging 0.018	BENIGN(0.065)
*SLC12A1*	c.1493C>T	Disease causing(0.9999)	Deleterious −3.39	Damaging 0.004	PROBABLY DAMAGING (1.000)
*CLCNKB*	c.229G>A	Disease causing(0.9999)	Deleterious −3.22	Damaging 0.031	PROBABLY DAMAGING (0.986)
*CLCNKB*	c.229G>C	Disease causing(0.9999)	Deleterious −4.12	Damaging 0.002	PROBABLY DAMAGING (0.991)

**FIGURE 2:**
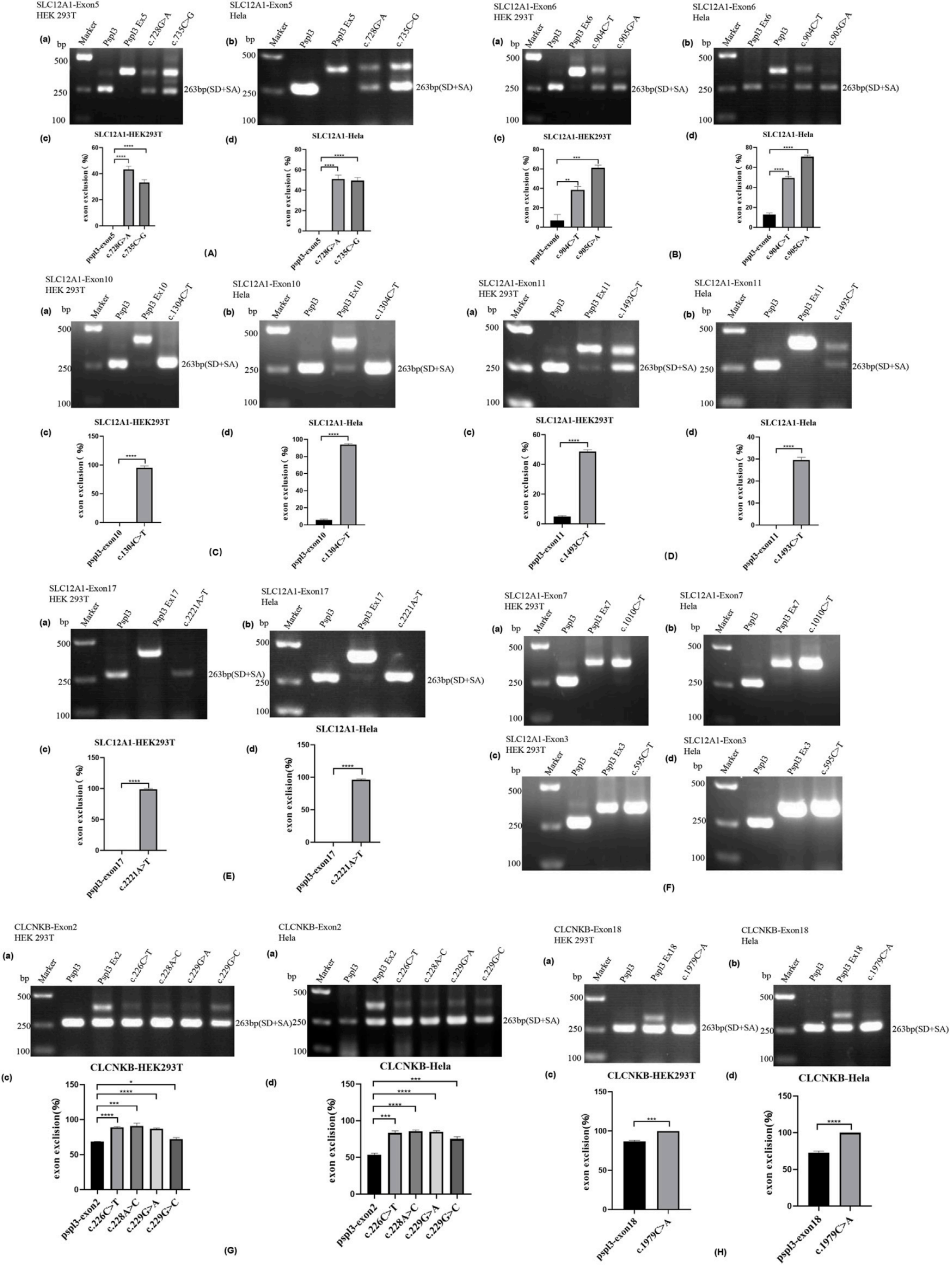
Agarose gel electrophoresis and statistical analysis of RT-PCR products in HEK 293T and Hela cells. Quantification of the splicing percentage was denoted by the percentage of exon exclusion (%) calculated as [lower band/(lower band + upper band)] × 100. Error bars represent SEM (n = 3). *p < 0.05; **p < 0.01; ***p < 0.001; ****p < 0.0001, unpaired Student’s t-test. **(A)** (a,b) Lane 1:marker;lane 2: pSPL3 (263 bp); Lane 3: pSPL3 Ex5 (405 bp); Lane 4: c.728G>A (405bp and 263 bp);Lane 5: c.735C>G(405bp and 263 bp). **(B)** (a,b) Lane1:marker;lane2: pSPL3 (263 bp); Lane 3: pSPL3 Ex6 (374 bp); Lane 4: c.904C>T (374bp and 263 bp);Lane 5: c.905G>A (374bp and 263 bp). **(C)** (a,b) Lane1:marker;lane2: pSPL3 (263 bp); Lane 3: pSPL3 Ex10 (415 bp); Lane 4: c.1304C>T (263 bp). **(D)** (a,b)Lane1:marker;lane2: pSPL3 (263 bp); Lane 3: pSPL3 Ex11 (415 bp); Lane 4: c.1493C>T (415bp and 263 bp). **(E)** (a,b)Lane1:marker;lane2: pSPL3 (263 bp); Lane 3: pSPL3 Ex17 (404 bp); Lane 4: c.2221A>T (263 bp). **(F)** (a,b) Lane1:marker;lane 2: pSPL3 (263 bp); Lane 3: pSPL3 Ex7 (375 bp); Lane 4: c.1010C>T (375bp). (c,d) Lane 1:marker;lane 2: pSPL3 (263 bp); Lane 3: pSPL3 Ex3 (339 bp); Lane 4: c.595C>T (339bp). **(G)** (a,b) Lane1:marker;lane2: pSPL3 (263 bp); Lane 3: pSPL3 Ex2 (392 bp and 263bp); Lane 4: c.226C>T (392 bp and 263bp);Lane 5: c.228A>C (392 bp and 263bp); Lane 6: c.229G>A (392bp and 263 bp).;Lane 7: c.229G>C (392bp and 263 bp). **(H)** (a,b)Lane 1:marker;lane 2: pSPL3 (263 bp); Lane 3: pSPL3 Ex18 (350 bp and 263bp); Lane 4: c.1979C>A (263 bp).

Different control minigenes were generated comprising *SLC12A1* WT sequences of exon3 (pSPL3-*SLC12A1* Ex3), exon5 (pSPL3-*SLC12A1* Ex5), exon6 (pSPL3-*SLC12A1* Ex6), exon7 (pSPL3-*SLC12A1* Ex7), exon10 (pSPL3-*SLC12A1* Ex10), exon11 (pSPL3-*SLC12A1* Ex11), exon17 (pSPL3-*SLC12A1* Ex17), and *CLCNKB* of exon2 (pSPL3-*CLCNKB* Ex2), and exon18 (pSPL3-*CLCNKB* Ex18), respectively. The amplified products were cloned into the pSPL3 expression plasmids and sequenced as described previously (Larijani et al., 2005). All the selected point variants were introduced into the corresponding minigenes by site-directed mutagenesis (Degiorgio et al., 2014). RT-PCR analysis indicated that some of them resulted in aberrant pre-mRNA splicing *in vitro* (Figure 2). What’s more, we conducted a predictive analysis of the functional consequences of exon skipping caused by point mutations, and details are listed in Table 3.

**TABLE 3:** Prediction of the functional consequences of exon skipping caused by point mutations in this study.

Mutation	Type	Gene	Sites of action	Splicing changes	Frameshift	Protein	References
c.728G>A	missense	*SLC12A1*	Splicing sites	partial skipping of exon5	p. Gly242Glyfs*2	truncated protein (NKCC2)	MacDougall et al. (2020)
c.735C>G	nonsense	*SLC12A1*	ESEs/ESSs	partial skipping of exon5	p. Gly242Glyfs*2	truncated protein (NKCC2)	Soudy et al. (2020)
c.904C>T, c.905G>A	missense	*SLC12A1*	ESEs/ESSs	partial skipping of exon6	in-frame deletions (codon 289–325)	37 aa loss in TMH4 and cytoplasmic topological domain (NKCC2)	Wang et al. (2021)
c.1304C>T	missense	*SLC12A1*	Splicing sites	complete skipping of exon10	p. Gly434Glyfs*58	truncated protein (NKCC2)	McGarvey et al. (2019)

c.1493C>T	missense	*SLC12A1*	ESEs/ESSs	partial skipping of exon11	in-frame deletions (codon 485–520)	36 aa loss in TMH8 and cytoplasmic topological domain (NKCC2)	McGarvey et al. (2019)
c.2221A>T	nonsense	*SLC12A1*	ESEs/ESSs	Complete skipping of exon17	in-frame deletions (codon 719–765)	47 aa loss between TMH11 and TMH12(NKCC2)	Soudy et al. (2020)
c.226C>T	nonsense	*CLCNKB*	Splicing sites	partial skipping of exon2	in-frame deletions (codon 34–77)	43 aa loss in TMH1(CIC-Kb)	MacDougall et al. (2020)
c.228A>C	synonymous	*CLCNKB*	Splicing sites	partial skipping of exon2	in-frame deletions (codon 34–77)	43 aa loss in TMH1(CIC-Kb)	Soudy et al. (2020)
c.229G>A,c.229G> C	missense	*CLCNKB*	Splicing sites	partial skipping of exon2	in-frame deletions (codon 34–77)	43 aa loss in TMH1(CIC-Kb)	McGarvey et al. (2019)
c.1979C>A	nonsense	*CLCNKB*	ESEs/ESSs	complete skipping of exon18	in-frame deletions (codon 644–672)	29 aa loss in cytoplasmic topological domain (CIC-Kb)	Famiglietti et al. (2014)

TMH, transmembrane helical; aa, amino acids.

### Splicing outcome of sequences variations of *SLC12A1*

#### Variants of exon5, 6, and 11 induced partial skipping of exons.

Variant c.728G>A (p. Gly243Glu) affected the G at the fourth nucleotide in exon 5 of *SLC12A1*. BDGP indicated that it may slightly reduce the score of the WT 3′ acceptor splice site from 0.99 to 0.98 (Table 1). Variants c.735C>G (p. Tyr245*), c.904C>T (p. Arg302Trp), c.905G>A (p. Arg302Gln) and c.1493C>T (p. Ala498Val) were all predicted to lead to inactivation of potential overlapping ESEs and generation of new ESSs with HSF(Table 1).

The result of the assay revealed that the splicing products detected in the mutant and WT minigenes were different. The control minigenes (pSPL3 Ex5, pSPL3 Ex6, pSPL3 Ex11) produced a unique band, whereas mutant minigenes (c.728G>A, c.735C>G, c.904C>T, c.905G>A and c.1493C>T) generated two different bands respectively (Figure 2A,B,D). Direct sequencing showed that the larger amplicons were the exon-included transcripts, while the smaller amplicons included only the 3′and 5′ pSPL3 exons.

Among these mutants, c.728G>A and c.735C>G prevented exon 5 inclusion, resulting in a subsequent frameshift from codon 243 and premature termination at the codon 244 in the mRNA of *SLC12A1*. The effect on the NKCC2 of connecting exons 4 and 6 would produce a truncated protein (Table3). The remaining mutants promoted exons skipping to different extent, leading to in-frame deletions. As a result, those mutant proteins would lose part of transmembrane helices (TMHs) and cytoplasmic topological domains (Table 3).

### Missense variants c.1304C>T (p. Ala435Val) result in complete skipping of exon 10

Variant c.1304C>T (p. Ala435Val) is located at position 4 in exon 10. This variation reduces the score of the 3′ acceptor splice site from 0.87 to 0.84 with BDGP analysis. The results of RT-PCR showed a unique product of 263 bp in mutant minigene and a larger band of 415 bp in WT minigene (Figure 2C-a,b). Sequencing analysis of all bands confirmed that the larger fragment corresponds to correctly spliced exons, while the smaller splice corresponds to a transcript without exon 10. Complete skipping of exon10 will lead to a frameshift alteration (from codon 434) and premature termination at the 492nd codon (Table 3), which was different from previous predictive results of polyphen-2 on the protein level (Table 2).

### Nonsense variant c.2221A>T (p. Lys741*) resulted in complete skipping of exon 17

Nonsense variant c.2221A>T (p. Lys741*) of the internal position of exon 17 in *SLC12A1* alters a AAG codon for Lys to a premature TAG stop codon. In silico analysis by HSF 3.1 software demonstrated that this variant not only disrupts four ESEs but also creates two ESSs. RT-PCR analysis results of minigenes after transfection indicated that the mutant and WT minigenes generated different sized products (Figure 2 E-a, b). The control minigene produced a band of 404bp, whereas the mutant minigene generated a unique product of 263 bp. Direct sequencing of two products showed that the larger amplicon was the transcript containing exon 17, and the smaller was the transcript excluding exon 17. The skipping of exon17 caused by c.2221A>T would lead to in-frame changes and loss of 47 amino acids in the transmembrane helical structure (Table 3).

### Variants in exon 3 and exon 7 did not alter pre-mRNA splicing

Variant c.595C>T (p. Arg199Cys), located at the 34th nucleotide position from the 3′end of exon 3, was predicted that this variation will lead to the disruption of one ESE site and generation of six new ESSs sites using bioinformatic tool HSF 3.1. Variant c.1010C>T (p. Ala337Val) identified at nucleotide position 35 of exon 7 were analyzed by HSF 3.1 to break two ESEs and create six novel ESSs. However, the RT-PCR products of these minigenes in *SLC12A1* matched in size with those generated by the respective WT constructs (Figure 2F). These were further confirmed by sequencing analysis. Thus, these variants did not influence pre-mRNA splicing.

### Splicing outcome of sequences variations of *CLCNKB*

#### Mutations caused skipping of exon 2 compared with the WT constructs

Nonsense variant c.226C>T (p. Arg76*), located at the fourth nucleotide from the downstream of exon 2. In silico prediction showed that this variant marginally reduced the score of the WT 5’splice donor site from 0.95 to 0.93. Variant c.228A>C (p.76Arg = ), as a synonymous variant located at the second nucleotide from the 3′end of exon 2, was predicted to decrease the score of the WT 5’splice donor site from 0.95 to 0.84 with BDGP and 8.55 to 5.41 with MxEntScan (Table 1). Putative *CLCNKB* missense mutations c.229G>A (p. Ala77Thr) and c.229G>C (p. Ala77Pro) involved the last nucleotide substitution of exon 2. Bioinformatics analysis with BDGP revealed that the score of the 5′donor site of intron 3 is 0.95, whereas it could not be analyzed by BDGP after variation (Table 1). The results of MxEntScan analysis indicated that both variations led to potential 5′donor splice site loss with the score from 8.55 to 2.25(G>A) and 3.78(G>C) respectively (Table 1).

RT-PCR analysis showed that all the mutant and WT minigenes produced two different lanes, located in 263bp and 392bp respectively (Figure 2 G-a, b). Analysis of cDNA prepared from HEK293T and Hela cells revealed that the amounts of the exon 2-skipping transcript of these mutants were significantly increased compared with those of the control plasmids (c.226C>T: 67.9 versus 87.8% in HEK293, 51.1 versus 85.7% in Hela; c.228A>C: 67.9 versus 91.0% in HEK293, 51.1 versus 85.6% in Hela; c.229G>A:67.9 versus 88.1% in HEK293, 51.1 versus 83.1% in Hela; c.229G>C:67.9 versus 74.4% in HEK293, 51.1 versus 78.4% in Hela) (Figure 2 G-c, d). The skipping of exon2 would lead to in-frame changes (Table 3). As a result, mutated CIC-Kb protein would lack the part of the transmembrane helices 1(TMH1).

### Nonsense variants c.1979C>A increased the amounts of the exon-excluded transcripts when compared with the WT-constructs

The nonsense variant c.1979C>A (p. Ser660*), located 50 bp upstream of exon18, was predicted that the variant not only eliminates five ESEs but also generates three novel ESSs by HSF3.1 analysis. As a result, two products of 263 bp (corresponding to defected transcripts) and 350 bp (corresponding to mature transcripts) were both found from the cDNA products of the WT minigenes in HEK 293T and Hela cells (Figure 2 H-a, b). Quantitative analysis of the PCR products revealed that the rate of defected transcripts to the full transcripts was 72.3% for HEK 293T and 70.7% for Hela cells, respectively (Figure 2 H-c, d). However, the unique products of 263bp were found in mutant minigenes (Figure 2 H-a, b), which suggested that this variant significantly enhanced pre-mRNA splicing. The exclusion of exon18 caused in-frame changes with 29 amino acids loss. Then, the mutated CIC-Kb protein would lose part of cytoplasmic topological domain (Table 3).

## Discussion

Pre-mRNA must undergo an RNA processing reaction called splicing that recognize exon-intron boundaries accurately to generate a mature translatable mRNA (Zhao et al., 2014). The splicing is carried out by the spliceosome, which interacts with relevant RNA splicing signals at exon-intron boundaries to catalyze the removal of noncoding introns and ligation of the exons. Moreover, splicing is dependent on a dense array of the motifs and intrinsic regulatory sequences, so mutations located at the cis-motifs have the potential to disrupt the splicing process and induce severe diseases. Currently, it is widely believed that the pathogenicity of variations should be validated at the mRNA level, because exonic variants outside the conserved GT-AG splice site could have been misclassified as nonsense, missense, or synonymous variants if only analyzed at the DNA level. Therefore analysis of RNA from patients is an ideal experimental method for the identification of potential splicing variants. Regrettably, RNA is difficult to obtain due to its poor stability and lower peripheral blood content. Alternatively, minigene splicing analysis has become an effective approach to assess the effect of variants on pre-mRNA splicing, which has been validated in our previous studies (Zhao et al., 2016; Zhang R. et al., 2018; Wang S. et al., 2020; Zhang et al., 2021).

Many point variants in *SLC12A1* and *CLCNKB* gene were previously considered as missense, nonsense, or synonymous mutations. However, as an increasing number of variants were verified to affect RNA splicing, we hypothesized that some variants in *SLC12A1* and *CLCNKB* may induce the alteration of pre-mRNA splicing as well. To further confirm whether the splicing efficiency was affected by the exonic variant, pSPL3 minigenes were constructed and transfected into cultured 293T and Hela cells respectively. As a result, 12 of the 14 candidate variants included in our study were found to cause whole or partial exon skipping (Figure 2).

Exonic juxtaposed sequence motifs, such as ESEs and ESSs, could regulate local exonic splicing by enhancing or silencing the identification of surrounding splice sites combined with diverse splicing factors (Shao et al., 2018). Thus, changes in proportion of ESEs/ESSs caused by exonic variants would affect the total strength of recognizing and using adjacent splice sites, resulting in abnormal splicing. In our case, missense variants c.904C>T (p. Arg302Trp), c.905G>A (p. Arg302Gln) and c.1493C>T (p. Ala498Val) were located in the internal region of exons 6 and 11 of *SLC12A1* respectively, and caused different degrees of exon skipping. Combined with the prediction results of HSF3.1, these variants significantly reduced the proportion of ESEs/ESSs, concomitant with the disruption of ESEs and generation of ESSs. The skipping of exon 6 or 11 would indue loss of amino acids without frameshift. Therefore, the mutant NKCC2 protein would lack corresponding parts of TMHs and cytoplasmic topological domains, which is essential for protein function.

As known, the common considered molecular pathogenic mechanism of nonsense variants was to induce nonsense-mediated mRNA decay or generate truncated protein (Nagy and Maquat, 1998; Littink et al., 2010). However, nonsense-related splicing changes (NAS), as another cellular RNA monitoring pathway that limited the number of mRNAs containing PTC, could be activated in the event of nonsense mutations (Mendell et al., 2002; Wang et al., 2002). This mechanism increased alternatively spliced mRNAs that removed deleterious PTCs and was therefore considered to be a positive post-transcriptional way (Lambert et al., 2020). Studies have shown that nonsense mutations might occasionally affect exon splicing by disturbing ESEs/ESSs and their interactors (Zhu et al., 2019; Abrahams et al., 2021). What’s more, the disruption of the reading frame in the transcript may trigger NAS (Chang et al., 2007). In our study, mutations c.735C>G (p. Tyr245*) and c.2221A>T (p. Lys741*) in *SLC12A1*, and c.1979C>A (p. Ser660*) in *CLCNKB* were predicted to result in disruption of ESEs and generation of ESSs by HSF3.1, and results confirmed that all of them induced abnormal splicing. Both c.2221A>T and c.1979C>A led to exclusion of corresponding exons and in-frame deletions, while variant c.735C>G caused the ligation of exons 4 and 6 in *SLC12A1* with a subsequent frameshift, resulting in the generation of truncated NKCC2 protein. In addition, nonsense variant c.226C>T (p. Arg76*), located close to the classical splicing site, affected the splicing of exon2 in *CLCNKB* probably by decreasing the recognition efficiency of the 5′ss DS or disrupting the reading frame. To sum up, the pathogenic mechanism and consequences of nonsense mutations should be emphasized from multiple aspects.

Variants c.728G>A (p. Gly243Glu) and c.1304C>T (p. Ala435Val) in *SLC12A1*, c.229G>A (p. Ala77Thr) and c.229G>C (p. Ala77Pro) in *CLCNKB*, were initially categorized as missense mutations, and all of them clustered around the classical splice sites (DS or AS). *In silico* analysis by BDGP indicated that all of them reduced the score of the 5′ or 3′ splice site to varying degrees, and results of splicing assays confirmed this presumption, leading to abnormal splicing. This was probably due to weakening of the strength of splice site recognition or creating of new splice sites resulted by these variants (Matos et al., 2018; Zhang et al., 2021). Variants c.728G>A and c.1304C>T were demonstrated to result in skipping of exon 5 and exon10 respectively, leading to frameshift alterations and premature terminations. Interestingly, unlike a “benign” “missense” variant predicted by the PolyPhen-2 software, c.1304C>T was actually a “detrimental” “splicing” variant. Therefore, it is necessary to assess the variants on the RNA level even if they are missenses. While variants c.229G>A (p. Ala77Thr) and c.229G>C (p. Ala77Pro) also disturb the normal splicing, as is presented in this splicing assay, with an increase of the proportion of exon 2-excluded transcripts compared with the WT clone. As a result, the skipping of exon 2 lead to an in-frame deletion and the loss of important domains of CIC-Kb. What’s more, we also confirmed that the synonymous substitution c.228A>C (p.76Arg = ) disturb the normal splicing process, which was consistent with a previous study (Wang C. et al., 2020).

Overall, 12 of 14 *SLC12A1* and *CLCNKB* single nucleotide alterations were confirmed to be splicing mutations via minigene splicing assays in this study. However, we can’t ignore the methodological limitations of a minigene strategy. Firstly, although it has served as an efficient tool for functional splicing analysis, it cannot explain the full pattern of splicing results. Secondly, the interaction of splicing factors in HEK 293T and Hela cells may have some difference from distal renal tubular cells, and the minigene assay may not completely simulate the *in vivo* situation. Thirdly, the transcription of mRNA may be affected by the secondary structure and nonsense-mediated decay (NMD), which was difficult for our minigene to capture. In addition, we didn’t examine the cell surface expression and activity of the NKCC2 and CIC-Kb. Thus, further studies are necessary for understanding the effects of splicing abnormalities on the pathogenesis of BS.

In our study, some presumed missense, nonsense, or synonymous variants were sorted and reclassified as splicing variants. We propose that more attention should be paid to their pathogenicity and potential therapeutic approaches. Up to now, antisense oligonucleotide (ASO) therapeutics targeting exon-skipping have shown potential therapeutic effects for various inherited disorders (Li et al., 2018). Future research and application of ASOs in urinary system are eagerly anticipated in the near future.

In conclusion, we have performed an extensive splicing analysis of exonic *SLC12A1* and *CLCNKB* mutations associated with BS using bioinformatics tools and minigenes. Results revealed that 12 previously presumed missense, nonsense or synonymous mutations altered pre-mRNA splicing. Variants c.735C>G, c.904C>T, c.905G>A, c.1493C>T, c.2221A>T in *SLC12A1*, and c.1979C>A in *CLCNKB* may disrupt ESEs and produce ESSs. Variants c.728G>A, c.1304C>T in *SLC12A1*, c.226C>T, c.228A>C, c.229G>A, and c.229G>C in *CLCNKB* caused exon splicing abnormalities probably by disturbing 3′ acceptor and 5′ donor splice site. This study highlights the necessity of assessing the impact of exonic mutations at the mRNA level in BS. The minigene is a valuable tool especially when the patients’ RNA or kidney specimens is unavailable or inaccessible.

## Data Availability

The original contributions presented in the study are included in the article/Supplementary Material, further inquiries can be directed to the corresponding authors.

## Supplementary material

Supplementary Figure S1 The direct sequencing results of point variants in *SLC12A1* and *CLCNKB*, the wild-type sequence is also shown.
