# Supplementary material for: Twelve exonic variants in the SLC12A1 and CLCNKB genes alter RNA splicing in a minigene assay
Source: Front Genet. 2022 Aug 25;13:961384. doi: 10.3389/fgene.2022.961384 (PMC9452827; doi:10.3389/fgene.2022.961384)
Supplement: Supplementary file 1 [file Table1.DOCX]

**Table S1** Primer sequences for amplifying exons.

| **Exons sequences** |
| --- |
| *SLC12A1* EXON3-PSPL3-F CCG CTCGAG AAGAGTGAACAAGGCCTGGG  *SLC12A1* EXON3-PSPL3-R CTA GCTAGC AGCAAAGTAAAAGGAGCCCA  *SLC12A1* EXON5-PSPL3-F CCG CTCGAG GGGCTAAGTTATTCACTGGG  *SLC12A1* EXON5-PSPL3-R CTA GCTAGC GATGCCAAGCCCAGGCTCCT  *SLC12A1* EXON6-PSPL3-F CCG CTCGAG ATATGGCCCCAGGTGTAATT  *SLC12A1* EXON6-PSPL3-R CTA GCTAGC AGCCCTGAATCATGACTGCC  *SLC12A1* EXON7-PSPL3-F CCG CTCGAG AGACCTATTTGCATGCCATT  *SLC12A1* EXON7-PSPL3-R CTA GCTAGC TGCTGGTGAAGTACTGGGTT  *SLC12A1* EXON10-PSPL3-F CCG CTCGAG AATGGCAGCCTGTTACAGAA  *SLC12A1* EXON10-PSPL3-R CTA GCTAGC AGTGCCAGGAGATGATCTCT  *SLC12A1* EXON11-PSPL3-F CCG CTCGAG CCTGGGCGATAGAGCGAGAC  *SLC12A1* EXON11-PSPL3-R CTA GCTAGC GAATCGATCTTGACTATGTG  *SLC12A1* EXON17-PSPL3-F CCG CTCGAG AATACCGCAGGTCATCTCCA  *SLC12A1* EXON17-PSPL3-R CTA GCTAGC GCTTTGCCTATACCCAGGAA  *CLCNKB* EXON2-PSPL3-F CCG CTCGAG TGCCCGGCCACTTATGTGAG  *CLCNKB* EXON2-PSPL3-R CTA GCTAGC CAGAGGAGGAAGAGCTCTTG  *CLCNKB* EXON18-PSPL3-F CCG CTCGAG ACATCAGGCCCCGCCCCTCT  *CLCNKB* EXON18-PSPL3-R CTA GCTAGC AGCCAGGCCCAGTGGCCAAT |

**Table S2** Primer sequences for introducing mutations into exons.

| **Mutations sequences** |
| --- |
| *SLC12A1*-EXON3-595-PSPL3-F TCATGCTCTTCATTTGCCTCTCCTGGATTG  *SLC12A1*-EXON3-595-PSPL3-R CAATCCAGGAGAGGCAAATGAAGAGCATGA  *SLC12A1*-EXON5-728-PSPL3-F TCTTGTTTCAGGTGAGGCCTACTATCTTAT  *SLC12A1*-EXON5-728-PSPL3-R ATAAGATAGTAGGCCTCACCTGAAACAAGA  *SLC12A1*-EXON5-735-PSPL3-F TCAGGTGGGGCCTAGTATCTTATTTCCAGA  *SLC12A1*-EXON5-735-PSPL3-R TCTGGAAATAAGATACTAGGCCCCACCTGA  *SLC12A1*-EXON6-904-PSPL3-F CAACCAATGACATCTGGATTATAGGCTCCA  *SLC12A1*-EXON6-904-PSPL3-R TGGAGCCTATAATCCAGATGTCATTGGTTG  *SLC12A1*-EXON6-905-PSPL3-F AACCAATGACATCCAGATTATAGGCTCCAT  *SLC12A1*-EXON6-905-PSPL3-R ATGGAGCCTATAATCTGGATGTCATTGGTT  *SLC12A1*-EXON7-1010-PSPL3-F CATTCTTCTAATTGTTATTGCAAACTTCTT  *SLC12A1*-EXON7-1010-PSPL3-R AAGAAGTTTGCAATAACAATTAGAAGAATG  *SLC12A1*-EXON10-1304-PSPL3-F TGTTTCCACAGGGGTCTGTGTGGTCCGAGA  *SLC12A1*-EXON10-1304-PSPL3-R TCTCGGACCACACAGACCCCTGTGGAAACA  *SLC12A1*-EXON11-1493-PSPL3-F CCCCCTCATCACTGTGGGAATCTTTTCTGC  *SLC12A1*-EXON11-1493-PSPL3-R GCAGAAAAGATTCCCACAGTGATGAGGGGG  *SLC12A1*-EXON17-2221-PSPL3-F AGGCCTGGCTTATATAGAACAAAATCAAGG  *SLC12A1*-EXON17-2221-PSPL3-R CCTTGATTTTGTTCTATATAAGCCAGGCCT  *CLCNKB*- EXON2-226-PSPL3-F GAGTGTGGTCTGAGGTAACC  *CLCNKB*- EXON2-226-PSPL3-R GGTTACCTCAGACCACACTC  *CLCNKB*- EXON2-228-PSPL3-F GTGTGGTCCGCGGTAACCCC  *CLCNKB*- EXON2-228-PSPL3-R GGGGTTACCGCGGACCACAC  *CLCNKB*- EXON2-229GA-PSPL3-F TGTGGTCCGAAGTAACCCCT  *CLCNKB*- EXON2-229GA-PSPL3-R AGGGGTTACTTCGGACCACA  *CLCNKB*- EXON2-229GC-PSPL3-F TGTGGTCCGACGTAACCCCT  *CLCNKB*- EXON2-229GC-PSPL3-R AGGGGTTACGTCGGACCACA  *CLCNKB*-EXON18-1979-PSPL3-F TTTGTGACGTAGCGGGGCAG  *CLCNKB*-EXON18-1979-PSPL3-R CTGCCCCGCTACGTCACAAA |
