# Supplementary figures and images for: Twelve exonic variants in the SLC12A1 and CLCNKB genes alter RNA splicing in a minigene assay
Source: Front Genet. 2022 Aug 25;13:961384. doi: 10.3389/fgene.2022.961384 (PMC9452827; doi:10.3389/fgene.2022.961384)

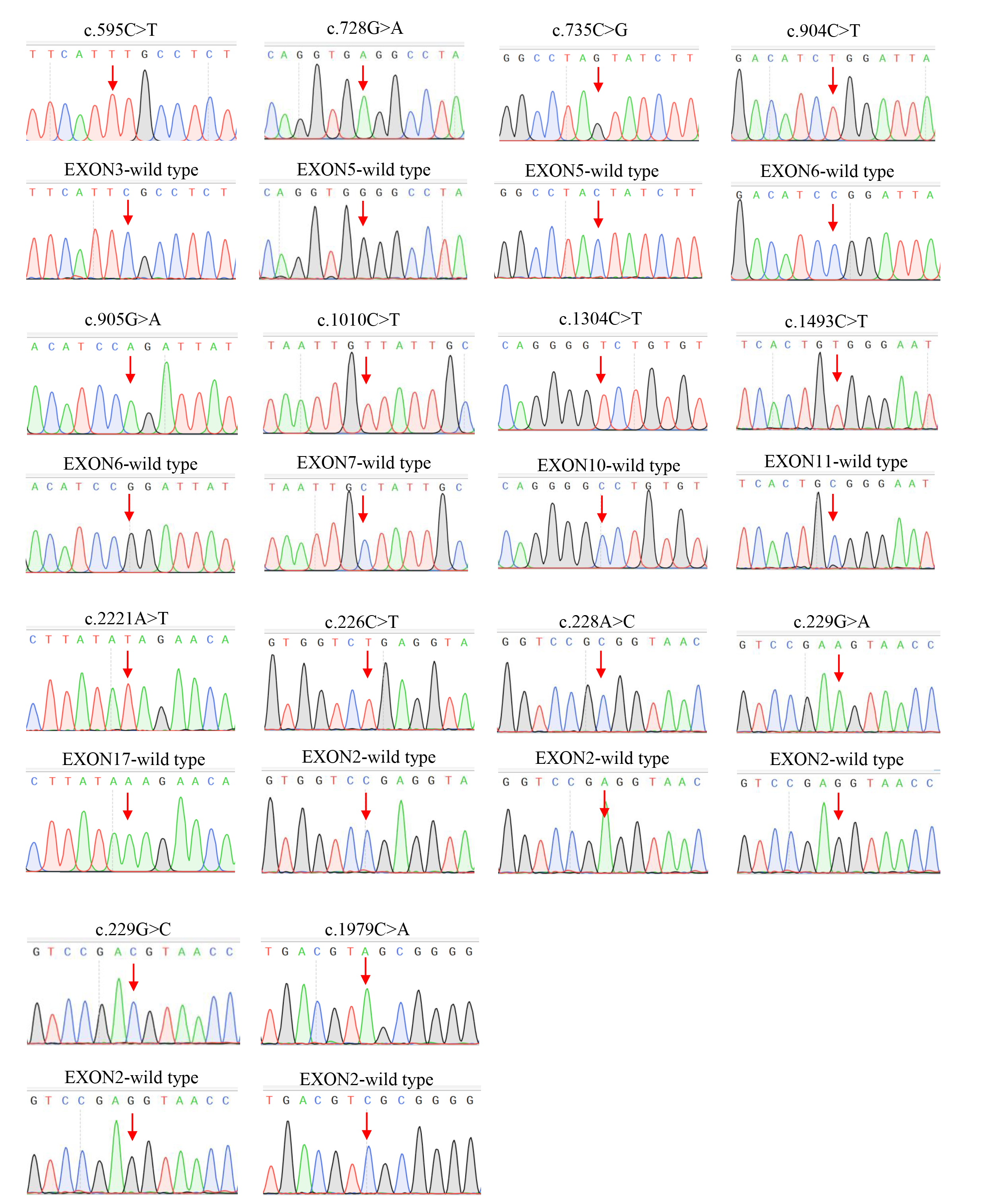

Supplement: Supplementary file 2 [file Image1.TIF]
